# Patterns of Mutual Associations Within the IL-12 Cytokine Family in Systemic Sclerosis Patients: A Pilot Study

**DOI:** 10.3390/biomedicines14102243

**Published:** 2026-10-03

**Authors:** Przemysław Kotyla, Olga Gumkowska-Sroka, Aleksander J. Owczarek, Kacper Kotyla, Jan Kurdybacha, Katarzyna Winsz-Szczotka

**Affiliations:** 1 Department of Rheumatology and Clinical Immunology, Medical University of Silesia, Voivodeship Hospital No 5, 41-200 Sosnowiec, Poland; oag@poczta.onet.pl; 2 Chair of Internal Medicine, Rheumatology and Clinical Immunology, Medical University of Silesia, 40-055 Katowice, Poland; 3 Health Promotion and Obesity Management Unit, Department of Pathophysiology, Faculty of Medical Sciences in Katowice, Medical University of Silesia, 40-752 Katowice, Poland; owczarek@paintbox.com.pl; 4 Department of Medical Biophysics, Medical University of Silesia Katowice, 40-752 Katowice, Poland; kacper.kotyla@gmail.com; 5 Students’ Society at the Department of Rheumatology and Clinical Immunology, Medical University of Silesia, Voivodeship Hospital No 5, 41-200 Sosnowiec, Poland; jkurdybacha@gmail.com; 6 Department of Practical Laboratory Medicine, Faculty of Pharmaceutical Sciences in Sosnowiec, Medical University of Silesia, Jedności 8, 41-200 Sosnowiec, Poland; winsz@sum.edu.pl

**Keywords:** systemic sclerosis, interleukin IL12 family, correlation study, pathogenesis of the disease

## Abstract

**Objectives:** This study aimed to investigate the associations among cytokines of the IL-12 family in patients with systemic sclerosis (SSc) and to evaluate their relationships with the pro-inflammatory cytokines TNF-α and IL-6, the latent form of TGF-β1 (LAP-bound, without prior sample activation), clinical parameters, and disease subtypes. **Methods:** Concentrations of IL-12p70, IL-12p40, IL-23, IL-27, IL-35, IL-39, TNF-α, and IL-6 were measured in serum, whereas latent TGF-β1 concentrations were determined in plasma obtained from 60 patients with systemic sclerosis (SSc) and 21 age- and sex-matched healthy controls. **Results:** Compared with healthy controls, patients with SSc exhibited significantly elevated levels of IL-12p40, IL-23, and IL-35, whereas IL-27, TNF-α, and TGF-β concentrations were significantly reduced. Correlation analysis revealed significant negative associations between latent TGF-β1 and IL-6, IL-27, and IL-35. Significant positive correlations were observed between TNF-α and IL-6, TNF-α and IL-12p40, IL-35 and IL-12p40, IL-23 and IL-12p40, and IL-12p40 with both IL-6 and IL-12p70. No significant associations were identified between the investigated cytokines and the Medsger severity score, patient age, anti-centromere antibody (ACA) status, anti-topoisomerase I antibody (Scl-70) status, the presence of pulmonary fibrosis, or diffusing capacity of the lungs for carbon monoxide (DLCO). In contrast, IL-35 levels demonstrated significant moderate negative correlations with both the modified Rodnan Skin Score (mRSS) (ρ = −0.35, *p* < 0.01) and disease duration (ρ = −0.30, *p* < 0.05). **Conclusions:** These findings may indicate the presence of a complex network of associations among IL-12 family cytokines in systemic sclerosis.

## 1. Introduction

Systemic sclerosis (SSc) is a connective tissue disease of unknown etiology and generally unfavourable prognosis, characterized by the coexistence of three major pathophysiological processes: autoimmune dysregulation, vasculopathy, and skin fibrosis [1]. Although skin fibrosis is a hallmark feature of the disease, internal organ involvement leading to end-stage organ failure contributes substantially to its poor prognosis [2]. Systemic sclerosis cannot be classified as a typical Th1- or Th2-dependent disease, as disease progression is accompanied by a characteristic shift from a Th1- to a Th2-dominant immune response [3]. These findings may have important clinical implications, supporting a stage-specific therapeutic approach. Anti-inflammatory interventions may be indicated during the early Th1-dominant inflammatory phase of the disease, whereas antifibrotic therapies may be more effective during the subsequent Th2-mediated fibrotic stage characterized by progressive tissue remodelling [4]. Therefore, it is crucial to determine the specific stage of the disease in each patient. In line with this concept, the roles of both pro- and anti-inflammatory cytokines may vary depending on the phase of disease progression, highlighting the dynamic immunopathological nature of systemic sclerosis.

The role of autoimmune responses in the pathophysiological cascade of systemic sclerosis, involving both the innate and adaptive arms of the immune system, is increasingly recognized as new evidence from experimental and clinical studies continues to emerge. In line with this concept, several pro- and anti-inflammatory cytokines have been investigated as potential and established pathophysiological factors in the pathogenesis of systemic sclerosis. To date, however, only a limited number of these cytokines have demonstrated a significant role in promoting autoimmunity and fibrosis. Among the latest, IL-6, TGF-β and IL-4 have proven their roles as important factors in SSc pathogenesis [5].

Less is known about IL-12 and other cytokines belonging to this family. Interest in IL-12 dates to the beginning of this century, when findings from observational studies indicated that both IL-12 and IL-23 are overexpressed in the tissues of patients with systemic sclerosis, and that their serum concentrations are elevated compared with healthy individuals [6]. The IL-12 family, initially classified as a group of pro-inflammatory and anti-inflammatory cytokines, comprises five members: IL-12, IL-23, IL-27, IL-35, and the recently identified IL-39.

Although IL-12 and IL-23 are recognized as prototypical proinflammatory cytokines and may potentially serve as key regulatory mediators in systemic sclerosis, data regarding their precise role in the disease remain limited. A major resurgence of interest in the IL-12 cytokine family occurred in 2007, when the heterodimer composed of Epstein–Barr virus-induced gene (EBI3) and p35 was identified as a biologically active cytokine and designated IL-35. This cytokine, secreted predominantly by regulatory T cells (Tregs) and regulatory B cells (Bregs), is characterized by potent immunosuppressive and anti-inflammatory properties. Recently, the latest member of the IL-12 cytokine family, IL-39, characterized by strong proinflammatory activity, has been described. In vitro studies have demonstrated that IL-39 is secreted by lipopolysaccharide (LPS)-stimulated B cells and antigen-presenting cells (APCs) [7]. Furthermore, IL-39 positively regulates the surface expression of intercellular adhesion molecule-1 (ICAM-1) and vascular cell adhesion molecule-1 (VCAM-1) on endothelial cells, thereby acting as a potent promoter of leukocyte trafficking and transendothelial migration [8].

However, to accurately define the role of a given cytokine within the pathophysiological milieu, both disease duration and, more importantly, the specific phase of the disease, should be taken into consideration. In systemic sclerosis, T helper (Th) cells and their associated cytokines are believed to orchestrate the major pathological processes underlying disease progression. Distinct phases of the disease may be further characterized by differential cytokine profiles detected in the serum and affected tissues of patients. It has been proposed that an imbalance among Th1, Th2, and Th17 cell subsets drives inflammation during the early stages of the disease, with a predominance of Th1- and Th17-related responses, followed by a shift toward a Th2-dominant immune response associated with progressive fibrosis in later stages of scleroderma [3]. Therefore, the role of IL-12 family members should be interpreted not only in the context of their pro- or anti-inflammatory properties but also concerning the specific phase of the disease. To date, the contribution of IL-12 family cytokines to the pathogenesis and progression of systemic sclerosis has not been comprehensively characterized.

Early studies conducted at the beginning of this century demonstrated significantly elevated serum IL-12 levels in patients with systemic sclerosis compared with healthy controls [6]. However, findings from subsequent investigations suggested a dynamic relationship between IL-12 concentrations and disease progression, particularly with regard to the development and progression of fibrosis [9]. Another member of the IL-12 cytokine family, IL-23, is a key component of the IL-23/Th17 axis, a pathway directly involved in driving T helper cell polarization toward a Th17 response and regulating the Th17/Treg balance, which is critically implicated in the progression of systemic sclerosis. Elevated serum IL-23 levels in patients with SSc have been shown to correlate with disease duration and the presence of interstitial lung disease [10]. Patients with systemic sclerosis exhibit significantly elevated IL-27 levels compared with healthy controls. These levels are positively correlated with the extent of skin and lung fibrosis. Moreover, increased IL-27 concentrations are associated with a more aggressive disease course and a higher risk of developing interstitial lung disease. At the level of cytokine crosstalk, IL-27 contributes to B-cell stimulation and immunoglobulin production, enhances IL-17 secretion by CD4+ T cells, and is involved in the regulation of fibroblast proliferation and collagen synthesis [11]. The anti-inflammatory cytokine IL-35 is upregulated in patients with systemic sclerosis; however, the degree of this upregulation varies significantly depending on the disease stage. Serum IL-35 concentrations are significantly upregulated in patients with an early systemic sclerosis pattern compared with those exhibiting late-stage disease patterns or healthy individuals. Moreover, patients with pulmonary fibrosis display higher IL-35 levels than those without fibrotic involvement [12,13]. Although individual cytokines belonging to the IL-12 family have been at least partially characterized, data regarding their mutual interactions, both within the IL-12 family and beyond it, remain limited. To address this gap, the present study was designed to evaluate the interplay among IL-12 family members, as well as their associations with key proinflammatory cytokines outside this family, namely IL-6 and TNF, with particular emphasis on disease activity and organ damage in systemic sclerosis.

## 2. Materials and Methods

### 2.1. Patients and Controls

This cross-sectional study included Caucasian patients with systemic sclerosis who fulfilled the 2013 American College of Rheumatology/European League Against Rheumatism (ACR/EULAR) classification criteria for SSc [14]. Participants were recruited from the local Systemic Sclerosis Database maintained by the Department of Rheumatology and Clinical Immunology at the Voivodeship Hospital in Sosnowiec, Poland. Additionally, 21 age- and sex-matched Caucasian healthy volunteers were enrolled as controls. According to the Medsger and LeRoy classification criteria, patients with SSc were further categorized into limited and diffuse cutaneous systemic sclerosis subsets [15]. The disease duration has been calculated from the first non-Raynaud symptom attributable to SSc [16]. The main exclusion criteria included previous or current treatment with biologic agents, including TNF inhibitors, IL-6 antagonists, co-stimulation inhibitors, and B-cell depletion therapy, CAR-T therapy; overlap syndromes (including, but not limited to, SSc/SLE, SSc/polymyositis, and SSc/rheumatoid arthritis); malignancy; prior bone marrow transplantation; and pregnancy and lactation. A detailed medical history and comprehensive physical examination were performed in all participants. Particular attention was paid to the onset, duration, and clinical course of the disease, with special emphasis on the onset of Raynaud’s phenomenon and the first non-Raynaud manifestation of SSc. Disease duration was calculated from the occurrence of the first non-Raynaud symptom, in accordance with commonly accepted methodology.

As part of the institutional database protocol, all patients with SSc routinely underwent high-resolution computed tomography (HRCT) of the chest as part of their standard clinical evaluation. For the present study, HRCT results were included if performed within the preceding 6 months; otherwise, the examination was repeated. In parallel with HRCT assessment, pulmonary function testing was performed. The evaluation included measurement of forced vital capacity (FVC), the ratio of forced expiratory volume in one second to forced vital capacity (FEV1/FVC), total lung capacity (TLC), and single-breath diffusing capacity for carbon monoxide corrected for hemoglobin (DLCO), in accordance with the American Thoracic Society/European Respiratory Society guidelines [17,18]. All results were expressed as percentages of predicted values.

Nailfold video-capillaroscopy was performed according to standard procedures using a Dino-Lite capillaroscope (Dino-Lite Europe, Almere, The Netherlands). Microvascular abnormalities were classified into early, active, and late scleroderma patterns [19].

As part of the standard of care for systemic sclerosis at our centre, routine transthoracic echocardiography has been performed, including estimation of systolic pulmonary arterial pressure (sPAP), calculated by adding right ventricular end-diastolic pressure to tricuspid regurgitation velocity (TRV), as well as assessment of left ventricular contractility by measuring ejection fraction (EF). Patients with a tricuspid regurgitation velocity (TRV) > 3.4 m/s were classified as having echocardiographic suspicion of pulmonary hypertension [20].

All patients were comprehensively characterized concerning serological status, including assessment of antinuclear antibodies (ANAs) and a systemic sclerosis-specific autoantibody profile (Euroimmun-Polska, Wrocław, Poland).

Clinical manifestations of systemic sclerosis included Raynaud’s phenomenon; cutaneous involvement characterized by pitting scars and active digital ulcers; and interstitial lung disease (ILD), defined by the presence of ground-glass opacities, subpleural reticulation with or without pleural irregularities, traction bronchiectasis, and/or honeycombing on routine high-resolution computed tomography (HRCT) scans. Oesophageal involvement was defined as the presence of persistent oesophageal dysphagia and/or oesophageal dilation detected on barium contrast radiography. Joint involvement was assessed clinically based on tender and swollen joint counts, while tendon friction rubs were also evaluated during physical examination. Renal involvement was defined by elevated serum creatinine levels and/or proteinuria exceeding 150 mg/day.

All patients were permitted to continue their standard medications, including immunosuppressive therapy for systemic sclerosis, provided that the administered doses did not exceed methotrexate 25 mg/week, azathioprine 200 mg/day, mycophenolate mofetil 2000 mg/day, or corticosteroids 10 mg/day. The use of other immunosuppressive therapies, including but not limited to cyclophosphamide, cyclosporine, tacrolimus, B-cell depletion therapy, and CAR-T therapy, was prohibited. Patients who had undergone bone marrow transplantation were also excluded from the study.

As part of the routine clinical assessment of patients with systemic sclerosis (SSc), the modified Rodnan Skin Score (mRSS) was determined according to the standard Rodnan skin thickness assessment [21].

Based on the collected clinical data, imaging studies, and laboratory findings, disease severity and disease activity were assessed using the Medsger Severity Scale and the revised EUSTAR activity index, respectively [22,23]—Table 1. The revised EUSTAR activity index was used to assess disease activity, with a score > 2.5 indicating active disease. Disease severity was evaluated using the Medsger Disease Severity Scale, which assesses the severity of involvement in nine organ systems on a 0–4 scale. As the Medsger scale measures cumulative organ damage and dysfunction rather than disease activity, no validated cut-off for active disease has been established.

### 2.2. Blood Sample Processing and Storage

Peripheral venous blood was collected from the cubital vein into tubes containing ethylenediaminetetraacetic acid (EDTA) for plasma preparation and into serum separator tubes for serum preparation. EDTA-anticoagulated blood samples were centrifuged for 10 min at 1500× *g* at 4 °C, and the plasma fraction was carefully transferred into sterile polypropylene tubes. Serum samples were allowed to clot completely at room temperature and were subsequently centrifuged for 10 min at 3000× *g* to separate the serum fraction from cellular components and clot residues.

The obtained plasma and serum samples were aliquoted into multiple portions to minimize repeated freeze–thaw cycles and stored at −80 °C until analysis. All samples were analyzed within 12 months after collection.

### 2.3. Biochemical Studies

Serum concentrations of IL-12p70, IL-12p40, IL-23, IL-27, IL-35, IL-39, IL-6 and TNF-α, as well as plasma concentrations of TGF-β1, were determined using commercially available quantitative sandwich enzyme-linked immunosorbent assay (ELISA) kits according to the manufacturers’ instructions. All assays were performed in 96-well microplates pre-coated with capture antibodies specific for the respective cytokines. Following incubation with samples, biotinylated detection antibodies and horseradish peroxidase (HRP)-conjugated streptavidin or avidin were added. Colour development was achieved using tetramethylbenzidine (TMB) substrate; the reaction was stopped with sulfuric acid, and absorbance was measured at 450 nm. Cytokine concentrations were calculated from standard curves generated for each assay.

Serum concentrations of IL-12p70, IL-12p40, IL-23, and IL-27 were measured using ELISA kits (Covalab, Bron, France). The IL-12p70 assay specifically recognized the biologically active p70 heterodimer without cross-reactivity with other human cytokines and had a detection range of 6.25–200 pg/mL with a sensitivity of 2.2 pg/mL (catalogue no. 30180). The IL-12p40 assay detected the p40 monomer, p40 homodimer, and the IL-12 (p35/p40) heterodimer, with a detection range of 62.5–2000 pg/mL and a sensitivity of 20 pg/mL (Covalab, Bron, France catalogue no. 99588). The IL-23 assay had a detection range of 156–5000 pg/mL and a sensitivity of 20 pg/mL (Covalab, Bron, France, catalogue no. 100411), whereas the IL-27 assay had a detection range of 31.25–1000 pg/mL with a sensitivity of 12.8 pg/mL (Covalab, Bron, France, catalogue no. 100412). For IL-27 values below 31 pg/mL, measurements were repeated with a new standard curve obtained by serial dilutions of the 1000 pg/mL standard to obtain concentrations from 31.25 pg/mL to 1.95 pg/mL (1000/500/250/125/62.5/31.25/15.62/7.81/3.90/1.95).

Serum concentrations of IL-35 and IL-39 were determined using sandwich ELISA kits (ELK Biotechnology, Wuhan, China). The IL-35 assay had a detection range of 15.63–1000 pg/mL and a sensitivity of 6 pg/mL (ELK Biotechnology, Wuhan, China catalogue no. ELK2745), whereas the IL-39 assay had a detection range of 7.82–500 pg/mL with a sensitivity of 3.2 pg/mL (ELK Biotechnology, Wuhan, China catalogue no. ELK9171).

Serum concentrations of IL-6 and TNF-α were measured using ELISA kits (LDN, Nordhorn, Germany). The IL-6 assay had a detection range of 23.3–2560 pg/mL and a sensitivity of 2 pg/mL (LDN, Nordhorn, Germany catalogue no. IL-E3200), whereas the TNF-α assay had a detection range of 6.8–518 pg/mL with a sensitivity of 0.7 pg/mL (LDN, Nordhorn, Germany catalogue no. IL-E3100).

#### 2.3.1. Measurement of Circulating TGF-β1

Plasma TGF-β1 concentrations were determined using the AssayMax™ Human LAP TGF-β1 ELISA Kit (catalogue no. ET3103-1; Assaypro LLC, St. Charles, MO, USA), which specifically quantifies the latency-associated peptide (LAP)-bound form of TGF-β1. As TGF-β1 circulates predominantly in a latent, LAP-associated form, this assay provides an assessment of the circulating latent TGF-β1 pool without prior sample activation. The assay is intended for the quantitative determination of human LAP-TGF-β1 in serum, plasma, and cell culture supernatants. TGF-β1 was assessed in plasma to minimize potential interference from platelet-derived TGF-β1.

The assay is based on a quantitative sandwich enzyme immunoassay. A murine monoclonal antibody specific for human LAP-TGF-β1 is pre-coated onto a 96-well microplate. LAP-TGF-β1 present in standards and samples is captured by the immobilized antibody and subsequently detected using a biotinylated polyclonal antibody specific for human LAP-TGF-β1. The bound detection antibody is subsequently recognized by a streptavidin–peroxidase conjugate. Following washing to remove unbound material, a peroxidase substrate was added, and the enzymatic reaction was terminated according to the manufacturer’s instructions. Absorbance was measured at 450 nm using a microplate reader. All samples were analyzed in duplicate.

The assay’s reported detection range was 0.031–2.0 ng/mL, with a lower limit of detection of 0.011 ng/mL. All measured TGF-β1 concentrations were above the reported detection limit.

For the other kits, when the concentrations of the analyzed metabolites were below the lowest calibrator concentration included in the reagent kit provided by the manufacturer, the calibration range was extended by performing serial dilutions of the calibrator standards. The accuracy of the obtained concentration values was subsequently verified using an internal standard (spike-recovery) approach, which involved adding a known amount of calibrator to the analyzed sample.

For IL-27, samples with concentrations below 31 pg/mL, corresponding to the lowest standard concentration in the kit, were reanalyzed using a newly generated calibration curve obtained by serial dilution of the 1000 pg/mL standard. The accuracy of the measurements was then verified by performing control measurements using the internal standard approach. Briefly, the IL-27 calibrator at a concentration of 62.5 pg/mL was mixed at a 1:1 volume ratio with selected samples whose concentrations fell below the quantifiable range of the manufacturer’s calibration curve, i.e., below 31.25 pg/mL. All subsequent analytical steps were performed in accordance with the manufacturer’s instructions.

The concentration obtained from the control measurement was then used to assess whether the sample concentration could be reliably quantified below the manufacturer’s stated lower limit. Values close to 31.25 pg/mL indicated that the sample concentration was outside the reliably quantifiable range of the assay. In contrast, values above 31.25 pg/mL indicated that concentrations below the manufacturer’s lowest calibrator could be reliably determined using the extended calibration curve. This approach was therefore used to establish the lower concentration limit for the analyzed metabolite.

For example, sample A, for which a concentration of 10.6 pg/mL had been determined using the extended calibration curve, was mixed at a 1:1 volume ratio with the IL-27 calibrator (62.5 pg/mL) and analyzed according to the manufacturer’s procedure. A concentration of 36.6 pg/mL was obtained. The concentration of the original sample was therefore calculated as follows:

2 × (36.6 − 31.25) = 2 × 5.35 = 10.7 pg/mL.

The resulting concentration was in close agreement with the value of 10.6 pg/mL obtained using the extended calibration curve. These control analyses confirmed the feasibility of extending the calibration curve to include standards at lower concentrations and, consequently, supported the validity of the concentration measurements obtained using the extended calibration range.

A similar procedure was applied to the determination of other analytes for which the measured concentrations fell outside the calibration range specified by the manufacturer.

The described procedure enabled the reliable quantification of analytes despite the limited amount of biological material available for analysis and the limited time available to perform measurements of all metabolites following sample thawing. Importantly, this approach also minimized the need for repeated freeze–thaw cycles, which could potentially compromise the integrity of the biological material provided for analysis.

#### 2.3.2. Sample Collection and Storage

Blood samples were collected into tubes containing EDTA as an anticoagulant. Plasma was separated by centrifugation at 3000× *g* for 10 min, after which the plasma was aliquoted and stored at −20 °C for up to 2 months. Repeated freeze–thaw cycles were avoided.

All kit components were stored according to the manufacturer’s recommendations. The conjugate and biotinylated antibody were stored at −20 °C, whereas the microplate, 10× diluent concentrate, wash buffer, stop solution, and chromogen substrate were stored at 2–8 °C. The standard was stored at 2–8 °C prior to reconstitution and at −20 °C after reconstitution.

#### 2.3.3. Assay Specificity

The manufacturer’s documentation does not provide data regarding potential cross-reactivity of the assay with IL-35 or IL-39. Available cross-reactivity data concern proteins from other species. Cross-reactivity with canine, bovine, mouse, rat, swine, and rabbit proteins was reported as undetectable, whereas cross-reactivity with monkey proteins was reported to be <50%. These data were considered in the interpretation of the assay results.

All measurements for a given analyte were performed within a single assay run on the same day, minimizing potential inter-assay variability.

### 2.4. Statistics

Statistical analyses were done using STATISTICA 13.0 PL (TIBCO Software Inc., Palo Alto, CA, USA) and R software version 4.6.1 (R Foundation for Statistical Computing, Vienna, Austria). The threshold for statistical significance was established at a *p*-value < 0.05, and all tests were two-tailed. Missing data were not imputed. Nominal and ordinal variables were presented as percentages, while interval data were shown as mean ± standard deviation for normally distributed data. For data with non-normal distribution, results were reported as median with interquartile range. The Shapiro–Wilk test and quantile–quantile (Q-Q) plots were used to assess data normality, and the Levene test was applied to check for homogeneity of variances. Group comparisons for interval data were made using one-way ANOVA with Tukey’s post hoc test or Kruskal–Wallis non-parametric analysis of variance. Nominal and ordinal variables were analyzed using the χ2 test. Correlations between variables were assessed using Spearman’s rank correlation coefficient. To assess factors related to cytokine levels, a generalized linear log-normal model was used. Moreover, we performed GLM log-normal models for each cytokine, adjusted for class, age, sex, time of disease, mRSS scale, and Fibrosis. The ROC curve analysis was carried out to find the cut-off of cytokine values that distinguish between SSc and the control group. Results were presented with sensitivity, specificity, and positive/negative predictive values with 95% confidence intervals (CIs). To account for the increased risk of false-positive findings arising from multiple pairwise correlation analyses among the measured cytokines (IL-6, TNF-α, IL-12p40, IL-35, and others as applicable), *p*-values were adjusted for multiple comparisons using the Benjamini–Hochberg or False Discovery Rate (FDR) procedure.

AI (Conceptviz-Pro) was used to generate the graphical abstract and Gemini AI 3.6 Flash for Figure 3.

### 2.5. Ethics

The study was designed and conducted in accordance with the principles of the Declaration of Helsinki. The study protocol was approved by the Bioethical Committee of the Medical University of Silesia, Poland (approval no. PCN/0022/KB1/16/I/20/21; issued on 4 May 2021). Written informed consent was obtained from all participants prior to the initiation of any study-related procedures.

## 3. Results

In this cross-sectional study, a total of 60 patients (70% women) with mean age 56.1 ± 12.0 years, diagnosed with systemic sclerosis according to the 2013 EULAR/ACR classification criteria, were identified and recruited from the local systemic sclerosis database maintained at the Clinical Department of Rheumatology and Clinical Immunology of the Voivodeship Hospital in Sosnowiec. Patients with systemic sclerosis were further classified as having either limited or diffuse systemic sclerosis according to the criteria proposed by LeRoy and Medsger. Based on this classification, 22 (36.7%) patients were identified as having limited systemic sclerosis and 38 (63.3%) as having diffuse systemic sclerosis. The median disease duration from first non-Raynaud symptoms was 8 years. The control group comprised 21 subjects with a mean age of 52 ± 8.5 years, including 10 men and 11 women.

All patients tested positive for antinuclear antibodies, as determined by immunofluorescence assay. Further evaluation using ELISA demonstrated positivity for anti-topoisomerase I (anti-Scl-70) antibodies in 36 (60%) patients, whereas 12 (20%) patients were positive for anticentromere antibodies (ACA). The remaining 12 patients were positive for anti-RNA polymerase III antibodies (4 patients), while 8 patients were triple-negative but exhibited the presence of associated antibodies, including anti-Ro52 (6 patients), anti-SSB (2 patients), and anti-Ku (2 patients) antibodies. Among patients with systemic sclerosis, videonailfold capillaroscopy revealed early, active, and late capillaroscopic patterns in 7, 27, and 16 patients, respectively. Patients with systemic sclerosis were characterized by active disease, with a mean revised EUSTAR activity index score of 3.38 ± 2.14 and a mean Medsger Severity Index score of 6.65 ± 3.81, calculated across all nine organ systems. Most patients had interstitial lung fibrosis (76.7%) and demonstrated a reduced diffusing capacity for carbon monoxide (DLCO), with a mean value of 71.6 ± 20.1% of the predicted value. We observed several significant differences between patients with limited and diffuse systemic sclerosis. Specifically, patients with limited systemic sclerosis had a shorter disease duration; less advanced skin involvement, as assessed by the modified Rodnan Skin Score; as well as lower disease severity and activity, reflected by lower Medsger Severity Index scores and revised EUSTAR activity index scores, respectively. Assessment of cytokines belonging to the IL-12 family revealed striking differences between patients and controls. Specifically, patients with systemic sclerosis (including both limited and diffuse subtypes) exhibited significantly higher serum levels of IL-12p40, IL-23, IL-35, and lower latent TGF-β1, compared to the control group.

Consistent with these findings, significant differences were also observed when the two disease subtypes were analyzed separately in comparison with healthy subjects. Patients with limited cutaneous systemic sclerosis were characterized by significantly elevated levels of IL-35, while exhibiting lower levels of TNF-α and latent TGF-β1 (in comparison to the control group). Similarly, patients with diffuse cutaneous systemic sclerosis demonstrated significantly elevated levels of IL-23, accompanied by lower levels of IL-27, TNF-α, and latent TGF-β1 in comparison to the control group. However, no significant differences in the analyzed interleukin levels were observed between the lcSSc and dcSSc groups. Clinical characteristics and comparison between patients and controls were summarized in Table 2.

### Correlation Study

**Figure 1:**
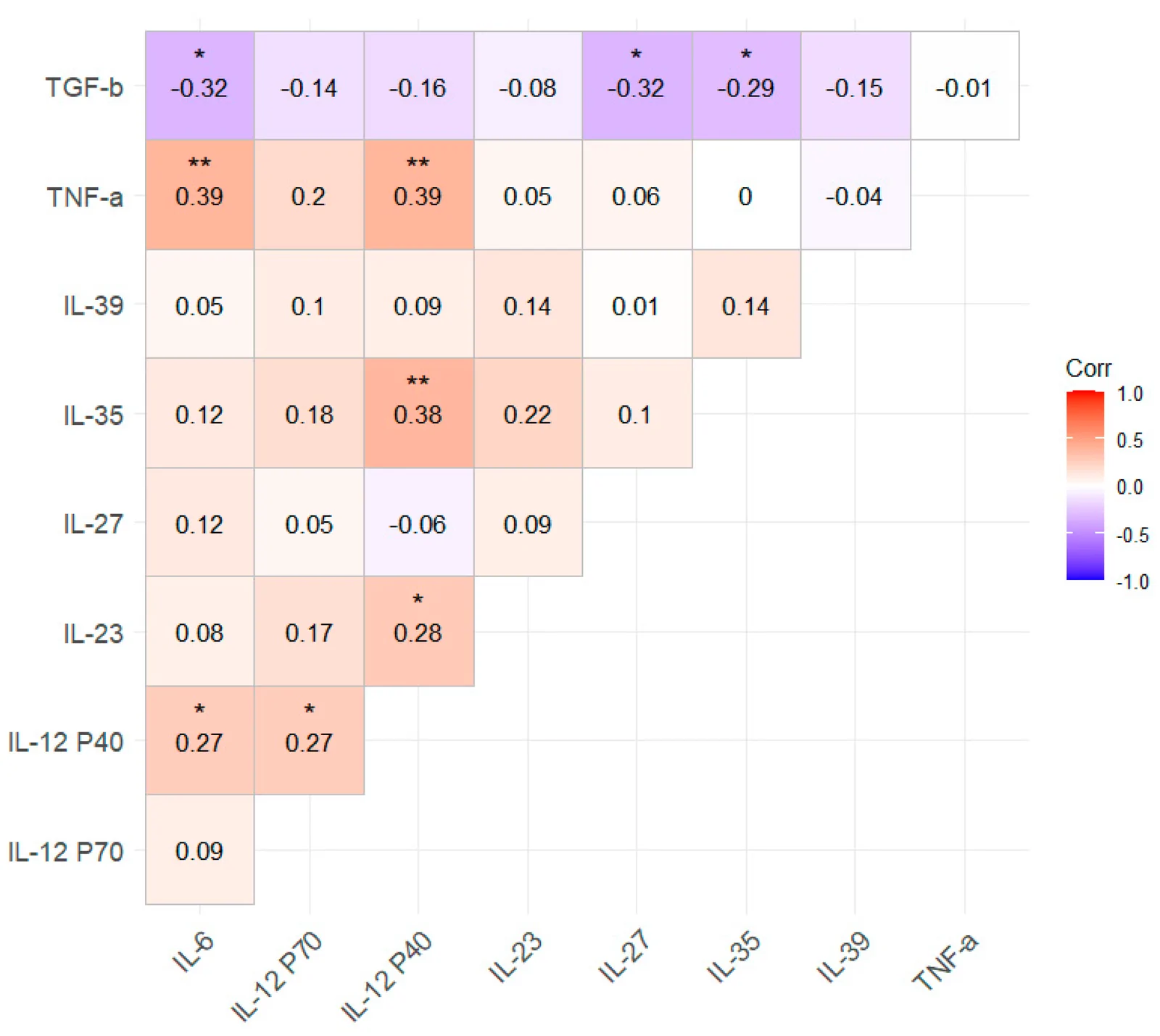
Spearman’s crude rank correlation coefficients between interleukins in the SSc group. * *p* < 0.05, ** *p* < 0.01.

To assess the mutual associations and hypothetical regulatory effects among cytokines, a correlation analysis was performed. Due to the exploratory nature of the analysis, *p*-values were not adjusted for multiple testing. According to the results obtained, significant negative correlations were observed between latent TGF-β1 and IL-6, IL-27, and IL-35. In contrast, significant positive correlations were identified between TNF-α and IL-6 as well as IL-12p40; between IL-35 and IL-12p40; between IL-23 and IL-12p40; and between IL-12p40 and both IL-6 and IL-12p70 (Figure 1). After FDR corrections for multiple testing, correlations between IL-6 and TNF-α, and between IL-12p40 and IL-35 and TNF-α remained significant (*p* < 0.05).

In the control group, there were moderate positive correlations between IL-27 and IL-12 p40 (ρ = 0.63; *p* < 0.01), IL-23 (ρ = 0.47; *p* < 0.05), as well as between TNF-α and IL-12 p40 (ρ = 0.54; *p* < 0.05), yet these were not significant after FDR corrections for multiple testing. In the first step, we performed a correlogram analysis to assess potential associations between the variables. Subsequently, generalized linear models (GLMs) with a log-normal distribution were fitted for each cytokine, adjusting for class, age, sex, disease duration, modified Rodnan Skin Score (mRSS), and fibrosis. None of these factors proved to be significant for any cytokine levels.

The next step of the study involved correlating cytokine levels with the clinical characteristics of the patients. In this part of the analysis, we demonstrated significant moderate negative correlations between IL-35 levels and both the modified Rodnan Skin Score (ρ = −0.35; *p* < 0.01) and disease duration (ρ = −0.30; *p* < 0.05). Furthermore, a significant moderate positive correlation was observed between the revised EUSTAR activity index and TNF-α levels (ρ = 0.31; *p* < 0.05).

Moreover, a moderate positive correlation between disease duration and latent TGF-β1 levels was noted (ρ = 0.39; *p* < 0.01). Furthermore, the IL-6 correlates positively with the Medsger scale (ρ = 0.28; *p* < 0.05) and revised EUSTAR activity index (ρ = 0.38; *p* < 0.01). In this study, cytokine levels did not correlate with other clinical characteristics, including seropositivity for ACA and anti-topoisomerase I antibodies, the presence of interstitial lung disease, or DLCO (%) values. Moreover, we have done GLM log-normal models for each cytokine adjusted for class, age, sex, time of disease, mRSS scale, and fibrosis. None of these factors proved to be significant for any cytokine levels. To demonstrate the lack of correlations between the analyzed variables, including age, sex, and the degree of fibrosis, we have included an example table presenting the results of our statistical analyses for IL-27 (Table 3).

No additional analysis was performed with respect to immunosuppressive treatment, as all patients included in the study received immunosuppressive therapy, as stated in the Patient Characteristics section. Therefore, treatment-related comparisons were not applicable.

**Figure 2:**
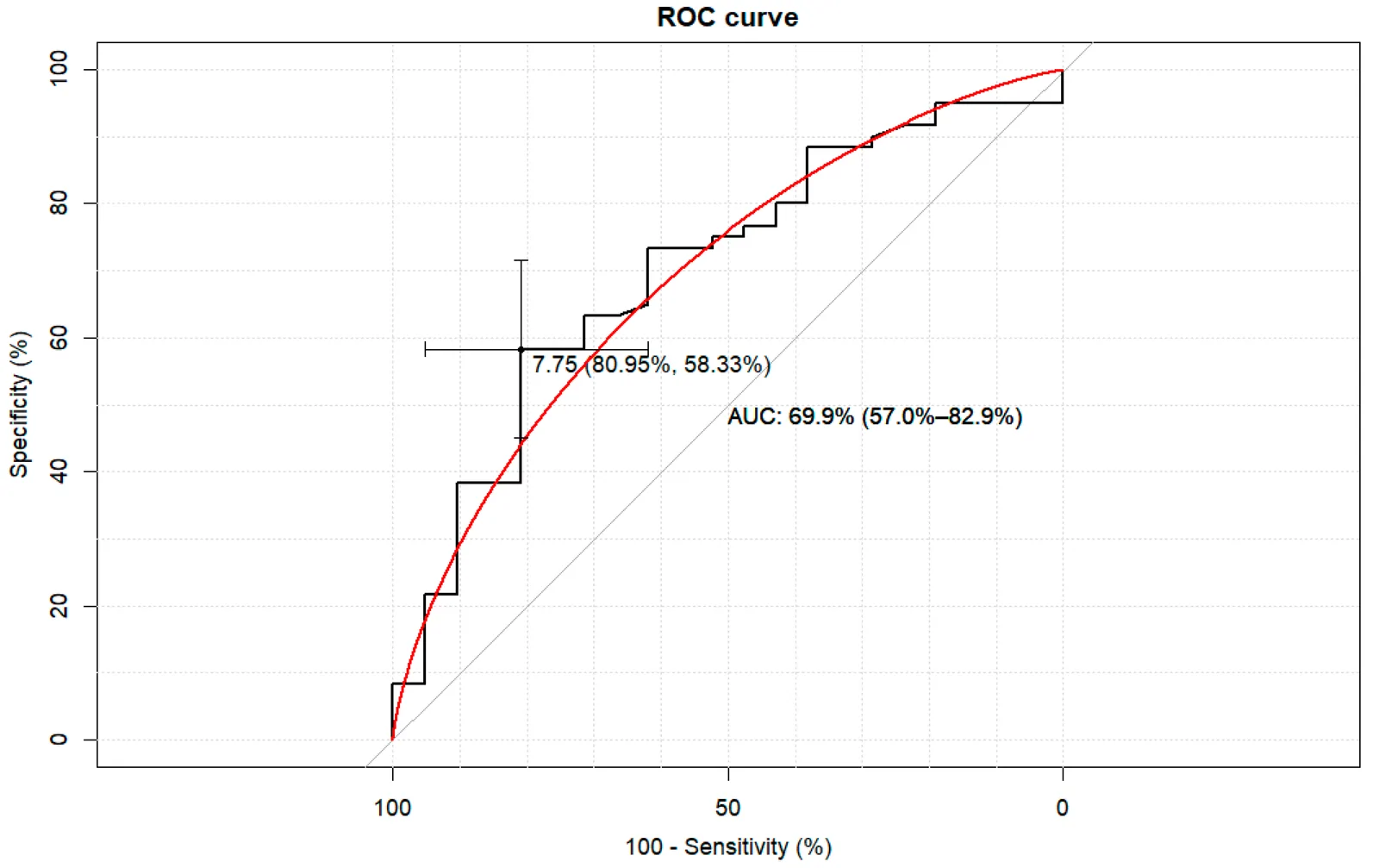
The Receiver Operating Characteristic (ROC) curve analysis for IL-27. The cut-off of 7.75 [pg/mL] distinguishes between SSc and the control group. The red line shows a binomial approximation of the ROC curve.

We also aimed to evaluate whether the assessment of IL-12 family cytokines could differentiate healthy subjects from patients with systemic sclerosis and to determine potential biomarker cut-off values for this distinction. ROC analysis demonstrated the potential, but poor-to-modest utility of IL-27 as a biomarker for identifying patients with systemic sclerosis based on reduced serum IL-27 levels, indicating its limited utility as a standalone diagnostic biomarker. (AUC: 69.9% (95% CI: 57.0–82.9%)). An IL-27 value below 7.75 (pg/mL) yielded a sensitivity of 58.3% (44.9–70.9%) and a specificity of 81.0% (58.1–94.6%) for distinguishing patients with systemic sclerosis from healthy controls (Figure 2). The positive/negative predictive values were as follows: 89.7% (75.8–97.1%) and 40.5% (25.6–56.7%), respectively.

## 4. Discussion

Cytokines belonging to the IL-12 family share a common structural composition but differ substantially regarding their physiological and pathophysiological functions, acting as either pro-inflammatory or anti-inflammatory mediators within the immunological milieu. The roles of IL-12 and IL-23 have been extensively associated with axial spondyloarthritis and psoriatic arthritis; however, their involvement in other connective tissue diseases in general, and in systemic sclerosis in particular, remains largely unexplored. In our study, we demonstrated that the levels of cytokines belonging to the IL-12 family differ significantly between patients with systemic sclerosis and healthy controls. Serum concentrations of IL-12p40, IL-23, and IL-35 were significantly elevated in patients with systemic sclerosis, whereas IL-27 levels were reduced and IL-12p70 concentrations remained unchanged. These findings contrast with the earlier observations reported by Sato, who demonstrated elevated IL-12 levels in systemic sclerosis and suggested their potential pathogenic role in internal organ involvement [6]. However, unlike our study, no separate analysis of IL-12 dimers was performed in that investigation, which may partially explain the discrepancies between the studies.

The IL-12 family plays a crucial role in the immune response, particularly through the regulation and polarization of T helper cells toward a Th1 immune response. In systemic sclerosis, alterations in IL-12 family cytokines may therefore reflect a shift from a predominantly fibrotic process toward an inflammatory response. Consequently, the role of IL-12 should be considered within the context of disease activity and fibrotic burden. Consistent with this notion, Matsushita et al. demonstrated that IL-12 overexpression was associated with the attenuation of skin fibrosis, supporting the hypothesis that IL-12 may exert protective effects against fibrotic tissue remodelling [9].

In our study, IL-12p40, but not IL-12p70, was found to be significantly elevated in the whole SSc group (but remained statistically insignificant when we analyzed dcSSc and lcSSc groups separately) compared with healthy controls. This finding likely reflects the specific stage of the disease, in which fibrotic processes predominate over active inflammation [24]. In the present study, we observed a positive correlation between IL-12p40 and IL-12p70 levels, which is consistent with the findings of Olleros et al., who reported co-expression of these cytokines in an animal model [25]. However, this association should be interpreted with caution. Although IL-12p40 constitutes an essential subunit of the bioactive IL-12p70 heterodimer, the observed correlation does not imply a direct stimulatory effect of IL-12p40 on IL-12p70 production. Rather, it is likely to reflect the coordinated regulation of IL-12 subunit expression by common upstream inflammatory signals. Consequently, increased production of the p40 subunit may occur concomitantly with the preserved synthesis of IL-12p70, resulting in the observed positive correlation. We may only speculate that elevated IL-12p40 levels may reflect increased activity of a negative-feedback mechanism limiting the IL-12-dependent proinflammatory response. It should be noted, however, that the immunoassay used does not distinguish between the p40 monomer, the p40 homodimer (considered an antagonistic form), and the IL-12 p35/p40 heterodimer (the biologically active IL-12 cytokine), but instead measures their combined concentration. Interpretation of elevated IL-12p40 as an indicator of a specific regulatory mechanism is therefore hypothetical and warrants further verification using methods able to differentiate between the individual p40 forms. Therefore, due to the design and limitations of our study, this observation needs to be verified in a specifically designed study. Nevertheless, this finding may help guide the direction of future research in this field. Another potent proinflammatory cytokine assessed in the present study, IL-23, was characterized by significantly elevated levels in patients with systemic sclerosis compared with healthy controls. Increased IL-23 concentrations have previously been reported by Hammad et al., and these findings are in strong agreement with our results, which demonstrated elevated IL-23 levels both in the overall systemic sclerosis cohort and, particularly, in patients with diffuse cutaneous systemic sclerosis (dcSSc).

In contrast to the study by Hammad et al., however, we did not observe any significant associations between IL-23 levels and clinical parameters. This discrepancy is likely attributable to differences in study populations. Patients included in the cohort studied by Hammad et al. were characterized by a shorter disease duration and more than twofold greater skin involvement, as reflected by higher mRSS [26].

The role of IL-23 in the immune response has been well established, as this cytokine plays a pivotal role in the differentiation and maintenance of the Th17 response, thereby exerting strong proinflammatory effects. However, the role of IL-23 in systemic sclerosis appears to be more complex. On the one hand, by promoting Th17-mediated immunity, IL-23 contributes to inflammation; on the other hand, it may also stimulate fibroblast activation and enhance type I collagen synthesis, thereby promoting fibrosis.

In the present study, IL-23 concentrations were significantly higher in patients with diffuse cutaneous systemic sclerosis and were positively correlated with IL-12p40 levels. Since IL-23 was not significantly associated with skin fibrosis, disease activity, or disease severity, the functional relevance of this observation remains unclear. It is possible that, similarly to IL-12p70, increased IL-12p40 expression may represent a compensatory or regulatory response to enhanced IL-23 production, as previously suggested [25]. This observation is particularly relevant because IL-12p40 is regarded as a natural antagonist of both IL-12p70 and IL-23, acting, at least in part, through competitive binding to the IL-12 receptor complex. Therefore, elevated IL-12p40 levels may simply reflect increased production of IL-12p70 and IL-23 and probably represent a negative-feedback mechanism aimed at attenuating cytokine signalling through receptor blockade [27,28].

Among the cytokines of the IL-12 family analyzed, only IL-27 was characterized by significantly lower levels in the overall systemic sclerosis cohort, as well as specifically in patients with diffuse cutaneous systemic sclerosis. This finding is in contrast to previously published studies, in which elevated IL-27 levels were positively associated with the extent of skin and pulmonary fibrosis, as well as with immunological abnormalities [29]. A more detailed comparison of the available data indicates that, in the Japanese cohort studied by Yoshizaki et al., more than 40% of patients exhibited normal or low IL-27 levels. Notably, this subgroup was characterized by a disease duration comparable to that observed in our cohort (7.2 vs. 8.0 years, respectively). IL-27 is a pleiotropic cytokine with context-dependent, biphasic activity, with both pro-inflammatory and anti-inflammatory effects described in different immunological settings. These properties suggest that IL-27 may be involved in complex immunological processes underlying systemic sclerosis and may potentially influence fibrotic pathways. However, the present study does not allow us to determine whether the observed IL-27 associations reflect a direct regulatory role or whether its biological effects differ according to disease stage or duration. The observed differences should therefore be interpreted as associations rather than evidence of a causal or stage-dependent regulatory mechanism. This may provide a possible context for comparison with the findings of Yoshizaki et al., particularly when analyses are restricted to patients with comparable disease duration [13].

In our study, a significant negative correlation between IL-27 and latent TGF-β1 levels was observed, suggesting a mutual association between these two cytokines. However, it should be emphasized that the assay used in this study measures LAP-bound latent TGF-β1 and not biologically active TGF-β1; therefore, the observed correlations cannot be directly interpreted as evidence of modulation of active TGF-β signalling or TGF-β-driven profibrotic activity. We may speculate that IL-27 may exert a regulatory role in modulating the balance between fibrosis and inflammation, potentially through the downregulation of profiles, levels and mediators such as TGF-β; however, this hypothesis would require confirmation using assays that specifically quantify active TGF-β1 or downstream TGF-β signalling markers (e.g., phospho-SMAD2/3). Moreover, ROC analysis demonstrated the potential, albeit modest, exploratory discrimination between cases and controls in this cohort based on reduced serum IL-27 levels. However, the discrepancies between our findings and previously published studies indicate that these results should be interpreted with caution, particularly in the context of disease duration and stage.

The findings regarding IL-35 are generally consistent with previous studies reporting increased expression or elevated circulating levels of this cytokine in systemic sclerosis. However, not all investigations have confirmed this observation, and some studies have reported discrepant results, suggesting potential heterogeneity among patient populations and differences in disease stage, clinical phenotype, or study design [30]. Elevated IL-35 levels, without significant differences between disease subtypes, are in close agreement with earlier findings. However, in contrast to our study, the authors did not observe any correlation between IL-35 levels and disease duration [12,31]. Similarly to the findings reported by Yayla et al., we did not observe any association between IL-35 levels and internal organ involvement. However, a significant negative correlation with the modified Rodnan Skin Score was observed, consistent with the findings previously reported by Yayla et al. [31,32]. We also observed marked differences between disease subtypes. When analyzed separately, only patients with limited cutaneous systemic sclerosis (lcSSc) exhibited significantly higher IL-35 concentrations than healthy controls (*p* < 0.01). This finding is consistent with the results reported by Osuna-Gómez et al., who likewise demonstrated elevated IL-35 levels in patients with the limited cutaneous form of the disease [30].

**Figure 3:**
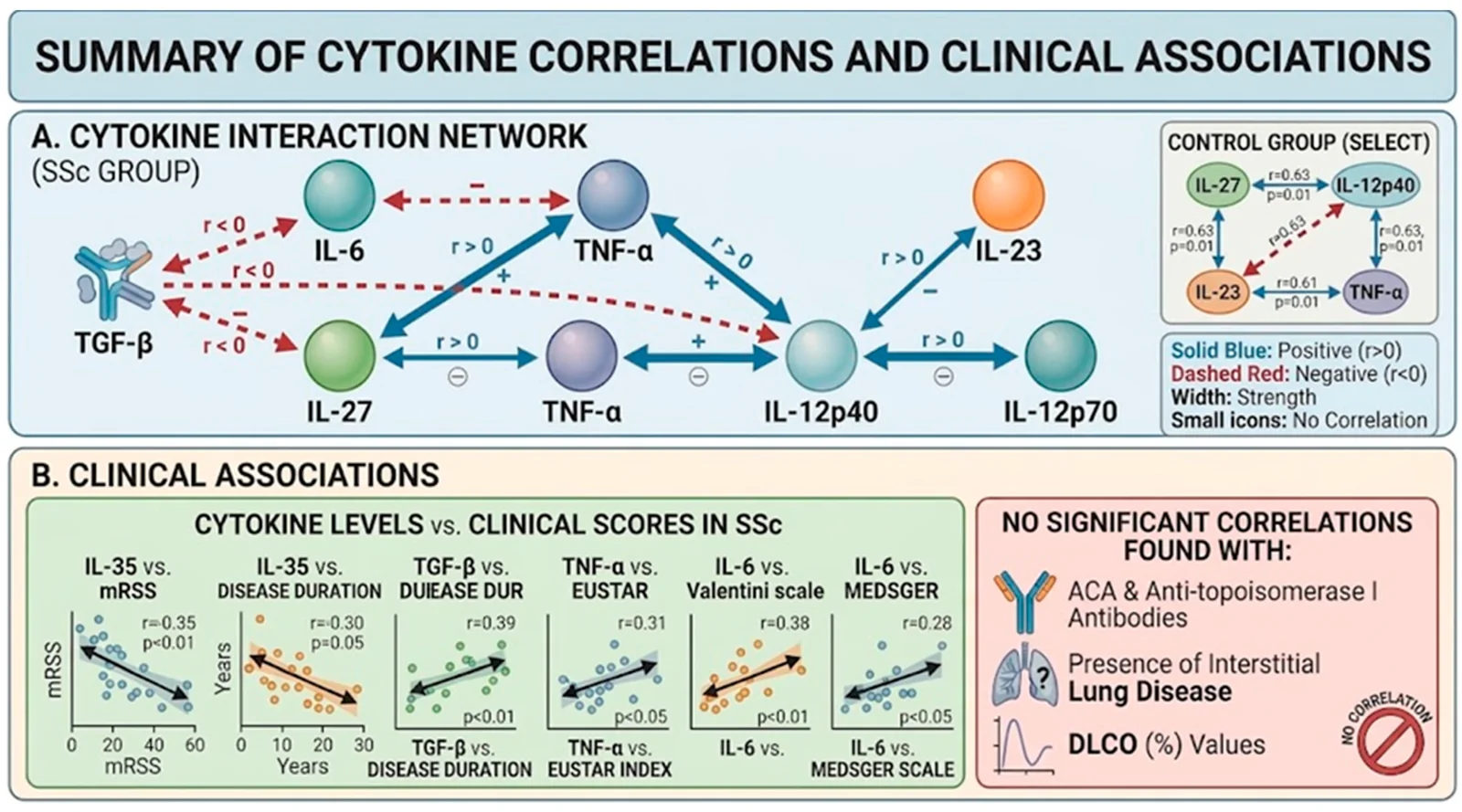
(**A**). Mutual association between cytokines of the IL-12 family, proinflammatory cytokines, and TGF-β. (**B**). Clinical association between cytokines and disease parameters (Created by Gemini). We can only speculate on the underlying mechanisms of this association. One possible explanation is that the observed effect is potentially mediated indirectly through increased production of the pro-inflammatory cytokines IL-12p70 and IL-23, which promote a shift in the immune response towards a pro-inflammatory phenotype, thereby attenuating fibrotic processes. Alternatively, IL-12p40 may act through the induction of IL-35, an immunoregulatory cytokine that has been proposed to suppress TGF-β-mediated fibrogenesis. Furthermore, IL-12p40 was positively associated with the production of TNF and IL-6, two key pro-inflammatory cytokines that may further promote immune activation and favour inflammation over fibrosis. In summary, our study demonstrated several mutual associations between the levels of IL-12 family cytokines, which may hypothetically suggest a mutual regulatory relationship. However, these results should be interpreted with caution, as our analysis relied solely on correlations between cytokine levels and did not investigate actual cytokine interactions at the receptor or gene product level. Thus, our findings should be regarded as a direction for future studies aimed at elucidating the real relationships between these cytokines, rather than as definitive evidence of such interactions.

Considering the potential role of IL-35 in the pathogenesis of systemic sclerosis, and considering the observed correlation analyses, it is plausible that elevated IL-35 levels may be characteristic of the early stages of the disease, with cytokine concentrations progressively declining over the course of disease duration and advancing skin fibrosis. This hypothesis may also account for the higher IL-35 levels observed in patients with limited cutaneous systemic sclerosis. A similar association between IL-35 levels and disease duration has previously been reported in another study [12]. This, however, should be interpreted with caution, as temporal changes in these relationships, if they occurred, cannot be inferred from our cross-sectional data. Further longitudinal and mechanistic studies are required to clarify the biological significance of these associations. In our study, a significant negative correlation between IL-35 and latent TGF-β1 levels was observed, suggesting a potential association between IL-35 and the latent TGF-β1 pool. However, since our assay quantifies LAP-bound latent TGF-β1 rather than biologically active TGF-β1, this observation cannot be directly interpreted as evidence of an inhibitory effect of IL-35 on active TGF-β signalling or TGF-β-associated profibrotic pathways. This interpretation is further supported by the negative correlation between IL-35 concentrations and the extent of skin involvement, as well as by significantly higher IL-35 levels observed in patients with the limited cutaneous form of systemic sclerosis. These findings are in contrast to those of Tomčík et al., who demonstrated a stimulatory effect of active TGF-β on dermal fibroblast activation in systemic sclerosis patients; however, we note that our measurement of latent TGF-β1 does not allow direct comparison with studies assessing active TGF-β signalling or its functional profibrotic effects [12]. Our results are also somewhat unexpected in the context of the generally described anti-inflammatory and profibrotic roles attributed to IL-35 [33]; however, published data in other autoimmune diseases have reported divergent or context-dependent effects of this cytokine, suggesting a more complex and disease-specific role [34]. Collectively, these findings may suggest a potential protective role of IL-35 against tissue fibrosis in systemic sclerosis. The negative correlation between IL-35 concentrations and disease duration may indicate that this protective mechanism diminishes with disease progression and the accumulation of irreversible fibrotic changes. This interpretation is supported by a recent Japanese study showing that IL-35 enhances IL-10^+^ regulatory B cell (Breg)-mediated immunoregulation and attenuates both inflammation and fibrosis in systemic sclerosis. Nevertheless, further studies are required to clarify the precise role of IL-35 in the pathogenesis and progression of fibrotic disease (Figure 3).

Moreover, as previously underlined, these mutual associations or interactions may reflect disease stage and are likely to undergo active changes throughout disease progression. Furthermore, it should be reiterated that the TGF-β1 concentrations reported in this study reflect the circulating latent (LAP-bound) TGF-β1 pool and do not directly measure biologically active TGF-β1 or active TGF-β signalling. Therefore, all correlations involving TGF-β1 should be interpreted as associations with the latent TGF-β1 pool, not as evidence of direct modulation of TGF-β-driven profibrotic activity. This could account for the discrepancies observed between studies, as well as the lack of correlation with clinical parameters in our population. To overcome this limitation, future studies conducted in a well-characterized and, more importantly, homogeneous, population of systemic sclerosis patients would be valuable.

## 5. Conclusions

In the present study, we demonstrated increased expression of several cytokines belonging to the IL-12 family, and their associations with the latent TGF-β1 pool, pro-inflammatory cytokines, and clinical parameters in patients with systemic sclerosis. We emphasize that TGF-β1 was measured in its latent (LAP-bound) form, and the reported correlations should not be interpreted as direct evidence of modulation of active TGF-β signalling or profibrotic activity.

Considering the structural complexity and biological interactions of these cytokines, it is plausible that regulation within the IL-12 cytokine network is mediated not solely through direct antagonistic effects, but also through more complex mechanistic pathways. Of particular interest is the concomitant correlation observed with other proinflammatory cytokines outside the IL-12 family, including IL-6 and TNF.

Findings from the study identify associations involving IL-35 and IL-27 in patients with SSc, suggesting that these cytokines may be involved in the complex immunological milieu of the disease. However, the present findings do not establish causal or direct regulatory relationships. Further longitudinal and mechanistic studies are needed to determine the functional significance of these associations and their potential evolution during disease progression.

## 6. Limitations of the Study

This study has several limitations. First, although we aimed to recruit a representative cohort of patients with systemic sclerosis, the relatively low prevalence of the disease limited the sample size, which may have reduced the statistical power of the analyses and introduced potential bias. Second, the assessment of the relationships among the investigated cytokines was based solely on correlation analyses. Consequently, these findings do not necessarily demonstrate direct biological interactions between cytokines, as could be established through mechanistic studies using isolated cells or other experimental models. Third, the analysis of cytokine profiles across different stages of the disease was based on indirect surrogate measures, including disease duration and the extent of skin involvement. A more accurate assessment of disease stage and immune polarization would require flow cytometric characterization of T-cell subsets to determine the relative predominance of Th1- and Th2-driven immune responses. As several cytokine concentration measurements fell below the assay’s detection limit, a dilution procedure was applied to reach the required detection threshold. However, this approach is not free from potential laboratory-related bias and may have influenced the results obtained in this study. Therefore, our findings should be interpreted with caution and considered preliminary rather than definitive.

A further limitation of this study is that the assay used to measure IL-12p40 detects the monomer, homodimer, and p35/p40 heterodimer collectively, without distinguishing between them. The results should therefore be interpreted as reflecting the total p40 pool rather than its free or antagonistic form specifically.

Finally, plasma TGF-β1 was measured using an ELISA that specifically quantifies the LAP-bound latent form of TGF-β1 without prior sample activation. This assay therefore does not assess biologically active TGF-β1, active TGF-β signalling, or profibrotic activity. Consequently, the observed correlations between cytokines and TGF-β1 should be interpreted as associations with the latent TGF-β1 pool rather than as direct evidence of the modulation of active TGF-β signalling or fibrogenic processes. Future studies employing assays that measure active TGF-β1 or downstream signalling markers (e.g., phospho-SMAD2/3) would be needed to clarify the functional significance of these associations.

## Figures and Tables

**Table 1:** Study group characteristics.

Variable	Value
Age [years]	56.1 ± 12.0
BMI [kg/m^2^]	24.8 ± 4.7
Disease duration [years]	8 (5; 12)
mRSS [pts]	11.3 ± 6.8
Medsger disease severity scale [pts]	6.65 ± 3.81
Revised EUSTAR activity index [pts]	3.38 ± 2.14
Lung fibrosis, N (%)	46 (76.7)
DLCO [%]	71.6 ± 20.1
Pulmonary arterial hypertension, N (%)	10 (16.7)
SCL-70 positive, N (%)	36 (60.0)
ACA positive, N (%)	12 (20.0)
Capilaroscopy, N (%)	
Early	7 (11.7)
Active	27 (45.0)
Late	16 (26.7)
Non-specific/normal	4 (6.7)
Not done/data not available	6 (10.0)

Mean ± standard deviation or median (lower quartile; upper quartile); BMI—Body Mass Index, mRSS—Modified Rodnan Skin Score, DLCO—diffusing capacity of the lungs for carbon monoxide, SCL-70—anti-topoisomerase I antibodies, ACA—anti-centromere antibodies.

**Table 2:** Comparison between the limited SSc, diffuse SSc, and the control group.

Variable	Control Group N = 21	SSc N = 60	Limited SSc N = 22	Diffuse SSc N = 38	*p*
IL-6 [pg/mL]	–	13.1 (2.0; 40.0)	2.90 (2.00, 25.70)	21.90 (2.00, 51.60) **	0.070
IL-12 P70 [pg/mL]	4.03 (3.29; 4.76)	5.24 (3.36; 6.57)	5.57 (4.46, 6.93)	4.67 (3.19, 5.88)	0.10
IL-12 P40 [pg/mL]	60.3 (33.1; 202.1)	135.8 (102.4; 180.8) *	134.4 (111.4, 155.6)	139.2 (84.2, 207.9)	<0.05
IL-23 [pg/mL]	31.7 (28.4; 31.9)	33.5 (30.8; 36.2) *	32.3 (30.2, 36.2)	34.1 (31.7, 37.3) *	<0.05
IL-27 [pg/mL]	9.51 (8.02; 13.10)	7.40 (5.07; 9.51) ^$^	7.73 (5.80, 9.04)	6.97 (4.48, 10.90) *	<0.05
Il_35 [pg/mL]	70.4 (9.7; 217.5)	209.6 (116.5; 364.4) ^$^	307.5 (135.5, 398.3) ^$^	148.9 (102.5, 283.9)	<0.01
IL-39 [pg/mL]	27.8 (7.4; 62.7)	46.9 (23.0; 108.4)	51.8 (21.4, 109.3)	46.8 (23.0, 106.5)	0.22
TNF-α [pg/mL]	10.50 (6.64; 21.50)	5.63 (3.85; 8.89) ^#^	4.69 (3.65, 7.69) ^$^	6.76 (3.85, 9.63) *	<0.05
TGF-β1 [ng/mL]	1.94 (1.63; 2.18)	1.15 (0.96; 1.44) ^#^	1.12 (0.88, 1.34) ^#^	1.21 (0.97, 1.52) ^#^	<0.001
Age [years]	54.8 ± 12.0	56.0 ± 11.7	54.9 ± 9.4	56.7 ± 13.4	0.55
Female, N (%)	11 (52.4)	42 (70)	17 (77.3)	25 (65.8)	0.23
BMI [kg/m^2^]	Not done	24.82 ± 4.7	23.7 ± 4.1	25.4 ± 4.9	0.18
Disease duration [years]	n/a	9.5 (4; 13)	6 (4; 9)	10 (7; 13)	<0.05
mRSS [pts]	n/a	9.54 ± 5.52	7.72 ± 4.65	13.03 ± 7.12	<0.01
Medsger disease severity scale [pts]	n/a	6.65 ± 3.81	5.18 ± 3.25	7.50 ± 3.89	<0.05
Revised EUSTAR activity index [pts]	n/a	3.38 ± 2.14	1.96 ± 1.15	4.08 ± 2.18	<0.001
Lung fibrosis, N (%)	n/a	46 (76.6)	12 (54.6)	34 (89.5)	<0.01
DLCO [%]	n/a	73.3 ±12.9	82.4 ± 20.0	66.0 ± 17.9	<0.01
Pulmonary arterial hypertension, N (%)	n/a	10 (16.6)	2 (9.1)	8 (21.0)	0.30
SCL-70 positive, N (%)	n/a	36 (60)	2 (10.5)	34 (94.4)	<0.001
ACA positive, N (%)	n/a	12 (63.2)	12 (63.2)	0	<0.001

Mean ± standard deviation or median (lower quartile; upper quartile); * *p* < 0.05, ^$^ *p* < 0.01, ^#^ *p* < 0.001 in comparison to the control group; ** *p* = 0.070 between dcSSc and lcSSc.

**Table 3:** Results of the multivariable log-normal GLM model for IL-27.

Parameter	β	SE (β)	e^β^	SE (e^β^)	CI	−95% CI	+95% CI	z-Value	*p*-Value
(Intercept)	2.385	0.373	10.862	4.049	0.95	5.232	22.552	6.399	<0.001
dSSc vs. lSSc	0.183	0.157	1.201	0.188	0.95	0.883	1.634	1.167	0.2434
Age [years]	−0.004	0.005	0.996	0.005	0.95	0.986	1.006	−0.741	0.4587
Female vs. Male	0.141	0.0141	1.151	0.162	0.95	0.874	1.517	1.001	0.3169
Disease duration [years]	−0.0135	0.009	0.987	0.009	0.95	0.969	1.004	−1.483	0.1381
mRSS [pts]	−0.008	0.009	0.991	0.009	0.95	0.973	1.010	−0.920	0.3573
Lung fibrosis yes vs. no	−0.272	0.0164	0.762	0.125	0.95	0.552	1.051	−1.659	0.0970

dSSc—diffused SSc, lSSc—limited SSc, mRSS—modified Rodnan Skin Score, SE—standard error, CI—confidence interval. β—regression coefficient, SE (β)—standard error of β, e^β^—multiplier (natural scale), SE (e^β^) 0 standard error of e^β^, z-value, *p*-value.

## Data Availability

The data presented in this study are available on request from the corresponding author due to local data protection policy.
